# High Expression of miR-218-5p in the Peripheral Blood Stream and Tumor Tissues of Pediatric Patients with Sarcomas

**DOI:** 10.1007/s10528-024-10873-8

**Published:** 2024-07-02

**Authors:** Fazilet Yıldız Özdenoğlu, Demet Akdeniz Ödemiş, Seda Kılıç Erciyas, Şeref Buğra Tunçer, Büşra Kurt Gültaşlar, Ahmet Salduz, Sema Büyükkapu, Necat Vakur Olgaç, Rejin Kebudi, Hülya Yazıcı

**Affiliations:** 1 Division of Cancer Genetics, Department of Basic Oncology, Oncology Institute, Istanbul University, 34093 Fatih, Istanbul, Türkiye; 2 Division of Cancer Genetics, Department of Basic Oncology, Health Sciences Institute, Istanbul University, 34093 Fatih, Istanbul, Türkiye; 3 Vocational School of Health Service, Medical LaboratortyTechniquies, İstanbul Okan University, Tuzla, Istanbul, Türkiye; 4 Istanbul Faculty of Medicine, Department of Orthopedics and Traumatology, Istanbul University, Istanbul, Türkiye; 5 Division of Pediatric Hematology and Oncology, Department of Clinical Oncology, Oncology Institute, Istanbul University, 34093 Fatih, Istanbul, Türkiye; 6 Faculty of Dentistry, Department of Oral Pathology, Istanbul University, 34093 Fatih, Istanbul, Türkiye; 7 Istanbul Arel Medical Faculty, Department of Medical Biology and Genetics, Istanbul Arel University, 34010 Zeytinburnu, Istanbul, Türkiye; 8 Turkey Cancer Institute, Health Institutes of Turkey, 34734 Kadıköy, Istanbul, Türkiye

**Keywords:** Ewing Sarcoma, Osteosarcoma, miR-218-5p, Expression, Peripheral Blood and Tumor Tissue

## Abstract

Sarcomas are malignant tumors that may metastasize and the course of the disease is highly aggressive in children and young adults. Because of the rare incidence of sarcomas and the heterogeneity of tumors, there is a need for non-invasive diagnostic and prognostic biomarkers in sarcomas. The aim of the study was to investigate the level of miR-218-5p in peripheral blood and tumor tissue samples of Ewing’s sarcoma, osteosarcoma, spindle cell sarcoma patients, and healthy controls, and assessed whether the corresponding molecule was a diagnostic and prognostic biomarker. The study was performed patients (*n* = 22) diagnosed and treated with Ewing’s sarcoma and osteosarcoma and in a control group of 22 healthy children who were matched for age, gender, and ethnicity with the patient group. The expression level of miR-218-5p in RNA samples from peripheral blood and tissue samples were analyzed using the RT-PCR and the expression level of miR-218-5p was evaluated by comparison with the levels in patients and healthy controls. The expression level of miR-218-5p was found to be statistically higher (3.33-fold, *p* = 0.006) in pediatric patients with sarcomas and when the target genes of miR-218-5p were investigated using the bioinformatics tools, the miR-218-5p was found as an important miRNA in cancer. In this study, the miR-218-5p was shown for the first time to have been highly expressed in the peripheral blood and tumor tissue of sarcoma patients. The results suggest that miR-218-5p can be used as a diagnostic and prognostic biomarker in sarcomas and will be evaluated as an important therapeutic target.

## Introduction

Ewing’s sarcoma and osteosarcoma are malignant tumors with a rather aggressive course in children, adolescent and young adults (Kim and Park 2016; Asif et al. 2018; Ross et al. 2013). Ewing’s sarcoma (ES) is an aggressive tumor that occurs in adolescents and young adults, accounting for 10–15% of all bone sarcomas (Ludwig 2008). Ewing’s sarcoma is a malignant small round cell tumor in which chromosomal translocations between genes of the E26 transformation specific (*ETS*) family, and *TET/FET* (*TLS/FUS, EWSR1 and TAF15*) family are frequently detected (Riggi and Stamenkoviç, 2007). The t (11; 22) (q24; q12) translocation is a common translocation in tumor. The *EWS* gene on chromosome 22 combines with the *FLI-1* gene on chromosome 11 to form a fusion protein, the Ewing sarcoma protein (Eaton et al. 2021).

The World Health Organization (WHO) has taken important steps to standardize and increase the accuracy of cancer diagnosis, especially for rare diseases such as soft tissue tumors and bone tumors, by publishing a classification in 2020. In the last 20 years, new genetic modifications, especially new fusion genes, have been discovered (Sbaraglia et al., 2021). The t (11; 22) (q24; q12) abnormality is detected in 90–95% of Ewing’s sarcoma patients. However, t (21; 22) (q2; q12) anomaly is detected in 5–10% of patients. Although the *EWS* gene is often found to fuse with the *FLI-1* gene, there are rare cases where the EWS gene fuses with the *ETV1, ERG* and *EA1F* genes. In some cases, *FUS,* substitutes the *EWS* gene, and the *FUS-ERG* fusion is formed with the translocation t (16; 21) (p11; q24) (Riggi et al. 2021). *FUS-ERG* shows strong CD99 membranous immunoreactivity. The forming of the fusion protein of *FUS* and *FEV* genes is also detected with a different translocation of the t (2; 16) translocation in Ewing’s Sarcoma (Ng et al. 2007). In addition, the presence of the *FUS-NFATC2* fusion was also detected with the RNA sequencing in the same disease (Pižem, 2020). These genetic features have led to the evaluation of the round cell sarcomas of soft tissue and bone in a separate category in the WHO classification of 2020. In addition to Ewing’s sarcoma, this new category also includes three subgroups that are clinically, pathologically, and molecularly different from Ewing’s sarcoma. These groups have been categorized as the round cell sarcomas associated with fusion forming EWSR1 (non-ETS fusions), the CIC (Capicua Transcriptional Repressor) rearrangement associated sarcomas, and BCOR (BCL-6 transcriptional co-repressor) associated sarcomas (Sbaraglia et al., 2021).

These tumors are small round cell tumors that usually occur in the bones and less commonly in the soft tissues (Ross et al. 2013). The characteristic immunohistochemical profile of this tumor varies for vimentin, CD99 (generally membranous), FLI1 (nuclear) and NKX2-2 (Dabbs, 2022). These tumors exhibit variable biological behaviors. This condition complicates the diagnosis, prognosis and treatment. Due to the similarities in the histological and immunohistochemical features of these tumors, differential diagnosis is difficult. Currently, genetic techniques such as FISH, PCR and new generation sequencing (NGS) play an important role in the final diagnosis (Asif et al. 2018). The disease most commonly occurs in the pelvis, followed by the femur, tibia, humerus, and scapula, but the disease can occur in almost any bone or soft tissue. Typically, patients present with pain and swelling in the affected area (Riggi and Stamenkoviç, 2007). Over the past 40 years, both local therapy and adjuvant chemotherapy with multiple agents have made significant advances in the treatment of localized disease, increasing the 5-year survival rate from 20% to over 70%; however, the recurrence rate is still high. Although many are localized, subclinical metastatic disease is present in almost all cases (Van Mater and Wagner 2019).

Osteosarcomas are the most common primary bone cancer in children, adolescent and young adults (Júnior et al., 2017; Gianferante et al. 2017; Gill and Gorlick 2021). The majority of tumors are high-grade and aggressive (Matsuoka et al. 2020). Osteosarcomas most commonly occur in the metaphysis of the long bones near the growth plates of the distal femur, proximal tibia, and proximal humerus (Bielack et al. 2002; Fletcher et al., 2002). Various drugs such as doxorubicin, ifosfamide, methotrexate, cisplatin, and epirubicin are used for treatment (Ayan et al., 2009). Metastases involving other organs particularly the lungs are detected in the disease (Júnior et al., 2017). The cytogenetic study results in osteosarcoma show that these tumors are aneuploidic. Since studies can also be carried out at the gene expression level today, osteosarcomas can be examined both with cytogenetic and molecular analyses. In addition, there is a wide spectrum of miRNA in osteosarcomas. The biomarkers obtained particularly in studies with circRNA and miRNA may be the cause of drug resistance. This situation once again reveals the importance and effective role of genetic diagnosis, especially at diagnosis and during chemotherapy (Özdenoğlu and Yazıcı, 2019).

miRNAs are the molecules of 19–25 nucleotides belonging to the group of non-coding RNA, which have critical roles in the regulation of gene expression in normal and pathological tissues (Parafioriti et al. 2016). miRNAs are processed by DROSHA, an RNA-specific ribonuclease enzyme complex from the primary transcripts known as pre-miRNAs (Cho 2010). Pre-miRNAs are converted into functional mature miRNA in the cytoplasm. This conversion is carried out by an endonuclease of DICER. Mature miRNAs regulate the protein production in the cell by binding to complementary target mRNAs for the RNA-induced silencing complex (RISC). The RISC perfectly matches with the target mRNAs to inhibit the protein expression through the separation and fragmentation of the mRNA. miRNAs have more than one target. Research show that miRNAs can act as oncogenes or tumor suppressor genes, and the changes in miRNA expressions are highly associated with various human cancers (Zhang et al. 2007; Yazici 2019). Various different miRNAs such as miR-34, miR-638, miR-107, miR-20b, miRNA-145-3p, miR-146b-5p, and miR-145 have been reported to be effective during the tumorigenesis of Ewing sarcoma and osteosarcoma (Cho 2010; Lin et al. 2015; Kawano et al. 2017; Wu et al. 2018; Qu et al. 2021; Tanoglu and Öztürk, 2021). miR-218 is one of the most well-known miRNAs and has been shown to inhibit progression in stomach cancer (Wang et al. 2017). miR-218-5p has been shown to act as a tumor suppressor in many human cancers such as hepatocellular carcinoma, bladder, mouth, stomach, and retinoblastoma cancers (Tu et al. 2014; Li et al. 2021; Li et al. 2020; Wang et al. 2020; Tang et al. 2016).

Emerging from mesenchymal tissues, bone and soft tissue tumors represent rare diseases characterized by a diverse array of histologic types and varying degrees of malignancy (Asano et al. 2019). Sarcomas, characterized by their heterogeneous nature, present a myriad of subtypes, posing diagnostic challenges despite advancements in identifying chromosomal translocations over the past three decades. While these diagnostic explorations have enhanced our understanding, the quest for reliable biomarkers persists. Recent studies delving into microRNAs in sarcomas offer promising avenues for addressing these challenges. Accumulating evidence on miRNAs in both patients with sarcomas and healthy cohorts underscores their potential for clinical utility. Furthermore, the identification of miRNAs serving as prognostic indicators opens avenues for miRNA-targeted therapies (Fujiwaro et al., 2014). Therefore, early diagnosis is important to improve the prognosis of patients with sarcomas. In a study conducted in 2022, it is shown that miR-218 serves as a significant inhibitor of tumor growth in glioma. Additionally, by suggesting that the mechanical properties of cells could provide a broad target area for drug intervention, they report that this offers potential opportunities for adjusting tumor cell behavior (Grabowska et al. 2022).

We investigated and evaluated the availability of the expression level of miR-218-5p as a diagnostic and prognostic biomarker in sarcomas in the present study. The expression level of miR-218-5p was investigated in the peripheral blood and tissue samples of cases consisting of 22 individuals with Ewing’s sarcoma and osteosarcoma and of 22 healthy control groups who were matched with the patient group in terms of age, sex and ethnicity.

## Materials and Methods

### Materials

The experimental group consisted of 22 sarcoma patients (Ewing’s sarcoma, Osteosarcoma, spindle cell sarcoma) who were admitted in the to the Division of the Pediatric Hematology-Oncology, Oncology Institute, Istanbul University, and of 22 healthy children who were matched with the patient group for age, sex, and ethnicity. The ethics committee approval of the study was obtained from the Clinical Research Ethics Committee in Istanbul University, Istanbul Faculty of Medicine dated 02.07.2020 with nu:74742. The parents of all children included in the study were informed about the study before the samples were taken, and samples were taken after the voluntary consent forms were signed. In the study, the appropriate concentrations of RNAs of the patient and healthy control groups that passed the quality control were used. The mean age of the experimental group was 12.1 ± 5 (5–18) years for the sarcoma group, and 10.5 ± 3.6 (4–18) years for the healthy control group. 22 patients were diagnosed before the age of 20 years, and only one patient who has a child with osteosarcoma and who was also diagnosed with osteosarcoma at the age of 38, also participated in the study as over the age of 20 years.

In addition, the blood/tumor tissue samples of a patient taken before treatment, during the surgery, and blood samples drawn in 3 months intervals after treatment were included in the study.

### Methods

First, the lymphocytes were isolated from the peripheral blood samples taken from the patient, and healthy control groups. The RNA samples were extracted from a lymphocyte pellet separated from peripheral blood using a commercial kit (ZYMO Research Quick-RNA Miniprep Plus Kit). The RNA was isolated from the tumor tissue samples using the same kit. The miRNA-specific cDNA synthesis was performed using a commercial kit of ID3EAL cDNA Synthesis System [MIRXES], for Real-Time PCR reaction. For the cDNA synthesis reaction, 5 µL RT-Buffer; 1 µL miRNA-218-5p primer; 1 µL RNU6-1 primer; reverse transcriptase 1 µL; and RNase/DNase-free water was added to obtain the final volume of 20 µL. The cDNA synthesis process was performed using the BioRad PCR device with incubation process performed at 42 °C for 30 min and then at 95 °C for 5 min. The Real-time PCR procedure was performed in the MIC qPCR Cycler device using 10µL SYBR Green; 2 µL qPCR assay primer; 5 µL diluted cDNA, and 3 µL of nuclease-free water, and the results were analyzed. The RNU6-1 molecule was used as a reference gene. The expression levels of each sample were calculated by the Ct (Cycle Threshold =) 2^−ΔΔCt^ method.

miR-218-5p and RNU6-1 primers were designed by the commercial company Mirxes Company, Inc. More information about the company can be found on company’s website. (http://miqe.gene-quantifcation.info/). The company’s website serves as a crucial platform for verification and reference, housing the gene sequences utilized in the research (Table 1).

**Table 1:** Sequence information of hsa-miR-218-5p and RNU6-1 reference genes in the study

Gene	Web page
Mature sequence of hsa-miR-218-5p (MIMAT0000275)	https://mircarta.cs.uni-saarland.de/mirna_view/256/
Precursor sequence hsa-miR-218-5p (MI0000294)	https://mirbase.org/hairpin/MI0000294?acc=MI0000294
RNU6-1 reference gene	https://www.ncbi.nlm.nih.gov/nuccore/NR_004394.1

## RESULTS

### Clinical Features of Patients

Information on the patient population included in the study and the biological material used in the patient population are given in Table 2. In the study, including peripheral blood from 22 patients, and peripheral blood samples from 22 healthy controls were used. The results of the distribution of the clinical characteristics of the patients with the pre-treatment miR-218-5p gene expression levels are given in Table 3. The median age of the patients was 12.1 ± 5 (5–18) years in the study. 22 patients were diagnosed before the age of 20 years, and only one patient who has a child with osteosarcoma and who was also diagnosed with osteosarcoma at the age of 38, also participated in the study as over the age of 20 years. The 38-year-old patient was not included in the median age. 15 patients were male, and 7 were female. The tumor diameters of the patients included in the study were between 0.3  cm and 19 cm, and the average tumor diameter of the patients was 8.3 cm for radiodiagnostic measurement. Metastasis was detected in three patients at diagnosis; however, no metastatic involvement was detected in 19 patients. In the evaluation of tumor location of the patients, it was seen that 3 of them were in the cranium, 5 of them were in the trunk, 1 of them was in the upper extremity and 14 of them were in the lower extremities. No statistical significance was detected in the comparisons between the expression levels of miR-218-5p, with the age of diagnosis (*p*: 0.705), sex difference (*p*: 0.245), tumor diameter (*p*: 0.649), metastatic status (*p*: 0.493), and tumor involvement sites (*p*: 0.694) of the patients (Table 4).

**Table 2:** Biological materials of the patients and control population used in the study

Used material of patients and controls	Peripheral blood			Tumor tissue	Total
	Controls	Patient			
		Before treatment	After treatment**		
Ewing sarcoma	0	13	0	1	14
Osteosarcoma	0	8+(1) *	2	1	11+(1)
Soft tissue sarcoma (spindle cell sarcoma)	0	1	0	0	1
Healthy controls	22	0	0	0	22
Total	22	22+(1)	2	2	48+(1)

*It belongs to the father of a patient with pediatric osteosarcoma who was diagnosed with osteosarcoma

**After 4th course chemotherapy

**Table 3:** Distribution of patients in accordance with the clinical features

Features	Patients *n* = 23(100%)	
Age	< 20	22 (95.7%)
	≥ 20	1 (4.3%)
Sex	Female	7 (30.5%)
	Male	16 (69.5%)
Tumor diameter	< 8 cm	10 (43.4%)
	≥ 8 cm	13 (47.6%)
Metastasis	Yes	3 (13.2%)
	No	20 (86.8%)
Tumor involvement region	Cranium	3 (13.05%)
	Trunk	5 (21.75%)
	Upper extremity	1 (4.35%)
	Lower extremity	14 (60.9%)

**Table 4:** The miR-218-5p expression level in sarcoma patients in accordance with the tumor region

Tumor location	miR-218-5p expression		Total	*p* value
	Decreased *n* (%)	Increased *n* (%)	Total *n* (%)	
Cranium	0 (0%)	3 (100%)	3 (13.05%)	0.694
Trunk	1 (20%)	4 (80%)	5 (21.75%)	
Upper extremity	0 (0%)	1 (100%)	1 (4.35%)	
Lower extremity	2 (14.28%)	12 (85.72%)	14 (60.9%)	
Total n (%)	3 (12.5%)	20 (87.5%)	23 (100%)	

The distribution of the tumor involvement region of the patients according to the expression level of miR-218-5p is given in Table 4. An increase was detected in the expression level of miR-218-5p with 80% and 85.72% ratio, respectively in most patients with tumors in the trunk and lower extremities, while a decrease was found in 20% and 14.28%, respectively. Although the number of patients in the other groups was small, there was an increase in the expression of miR-218-5p in all patients in the small groups (Table 4).

### Comparison of miR-218-5p Expression Level Between Whole Sarcoma Patients and Healthy Control Group at the Time of Diagnosis

The microRNA expression differences were evaluated using the 2^−ΔΔCt^ formula.

2^−ΔΔCt^ is used to calculate gene expressions.

$$\Delta {\text{Ct}} = {\text{Ct}}\left( {\text{Target gene}} \right) - {\text{Ct}}\left( {\text{Reference gene}} \right)$$

$$\Delta \Delta {\text{Ct}} = \Delta {\text{Ct}}\left( {{\text{Patient}}} \right) - \Delta {\text{Ct}}\left( {\text{Average of Healthy Controls}} \right)$$

$$2^{{\left( { - \Delta \Delta {\text{Ct}}} \right)}} = 2^{{ - \left[ {\Delta {\text{Ct}}\left( {{\text{Patient}}} \right) - \Delta {\text{Ct}}\left( {{\text{Average}}\;{\text{of}}\;{\text{Healthy}}\;{\text{Controls}}} \right)} \right]}}$$

According to this method, the results were determined based on the Ct values, which represent the first significant increase in the amount of PCR products. Ct (cycle threshold), is the cycle at which the system begins to detect an increase in fluorescence and the PCR product starts to increase exponentially in the logarithmic and linear phases. Ct levels are inversely proportional to the amount of target nucleic acid in the sample.

**Fig. 1:**
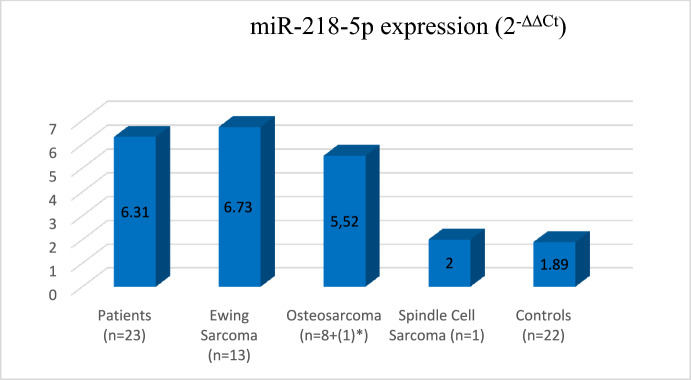
The miR-218-5p expression levels of all patients and healthy controls (patients *n* = 23 (Ewing sarcoma *n* = 13; Osteosarcoma *n* = 8+(1)*; spindle cell sarcoma *n* = 1)). * It belongs to the father of a patient with pediatric osteosarcoma who was diagnosed with osteosarcoma

In this method, the ΔCt values of the target gene and the reference gene were separately calculated for the patient, and control group. Then, the mean ΔCt value of the healthy group was determined from the ΔCt value of each patient sample and the ΔΔCt value of the sample of each patient was found. The increases and decreases in the expression levels of miR-218-5p belonging to the patient group compared to the control group were determined in accordance with the formula 2^−ΔΔCt^ having the RNU6-1 as the reference gene expression. The graphs showing the 2^−ΔΔCt^ increase or decrease of the expression levels of sarcoma patients compared to the levels in the healthy group are given in Fig. 1. Considering the Fold & Change (|FC|≥ 2) value of the patient data, the gene expression levels were found to have increased higher than two-fold. The comparison of the level of miR-218-5p gene expression in all patients compared to the levels in the healthy control group was found to have increased 3.33-fold at the time of diagnosis. In the comparison between the group of Ewing sarcoma patients and the group of control, the miR-218-5p expression level was increased by 3.56-fold while in comparison between patients with osteosarcoma and the control group, the level of miR-218-5p expression was increased 2.92 times (Fig. 1). The Mann–Whitney U test was performed based on the 2^−∆∆Ct^ values of the patient and control groups. The Mann–Whitney U test showed that the expression level of miR-218-5p was found statistically significant in the patient group compared with the expression levels in the healthy controls (*p* = 0.006). Additionally, since the sample size was below 30, a non-parametric test, the Mann–Whitney *U* test, was used for data analysis. Furthermore, ROC (Receiver Operator Characteristics Curve) analysis was conducted to reveal the diagnostic power of the candidate biomarker miRNA.

**Fig. 2:**
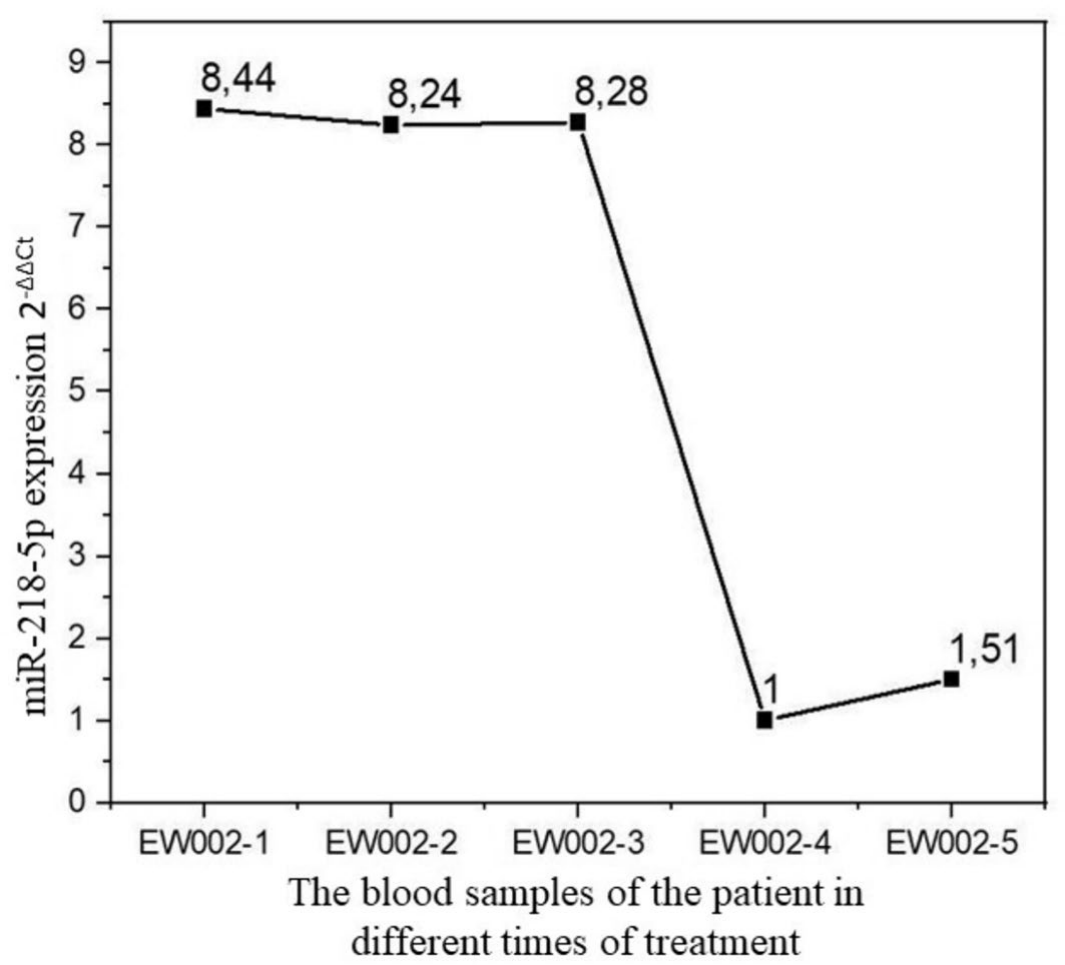
The miR-218-5p gene expression values in the peripheral blood and tumor tissue samples of the osteosarcoma patient: EW002_1: The peripheral blood drawn at the time of diagnosis; EW002_2: Tumor tissue dissected during surgery; EW002_3: The peripheral blood drawn during surgery; EW002_4: The peripheral blood drawn after the 1st course of chemotherapy; EW002_5: The peripheral blood drawn after the 4th course of chemotherapy (After 1st selection chemotherapy 1)

Only one patient who was female and 16 years old at diagnosis agreed to donate blood for the study at the time of diagnosis and at different stages of treatment. For this reason, peripheral blood and tumor tissue samples of a patient were studied before the treatment, during the operation, after the 1st and 4th cycles of the chemotherapy. It was determined that the expression levels of miR-218-5p in the peripheral blood sample taken during the diagnosis, that was, before the treatment, and in the blood and tumor tissue taken during the operation were extremely close to each other. It was observed that miR218-5p expression in peripheral blood samples taken before treatment, after 1st and 4th cycles of chemotherapy decreased almost 8.44 folds. This situation at different stages of treatment was given in the diagram in Fig. 2. The miR-218-5p gene expression level in the tumor tissue and peripheral blood samples taken during the surgery were found close to the value detected at diagnosis as 8.24 and 8.28, respectively. The expression level of miR-218-5p was measured as 1 and 1.51 after 1st and 4th chemotherapy, respectively for the patient (Fig. 2).

**Fig. 3:**
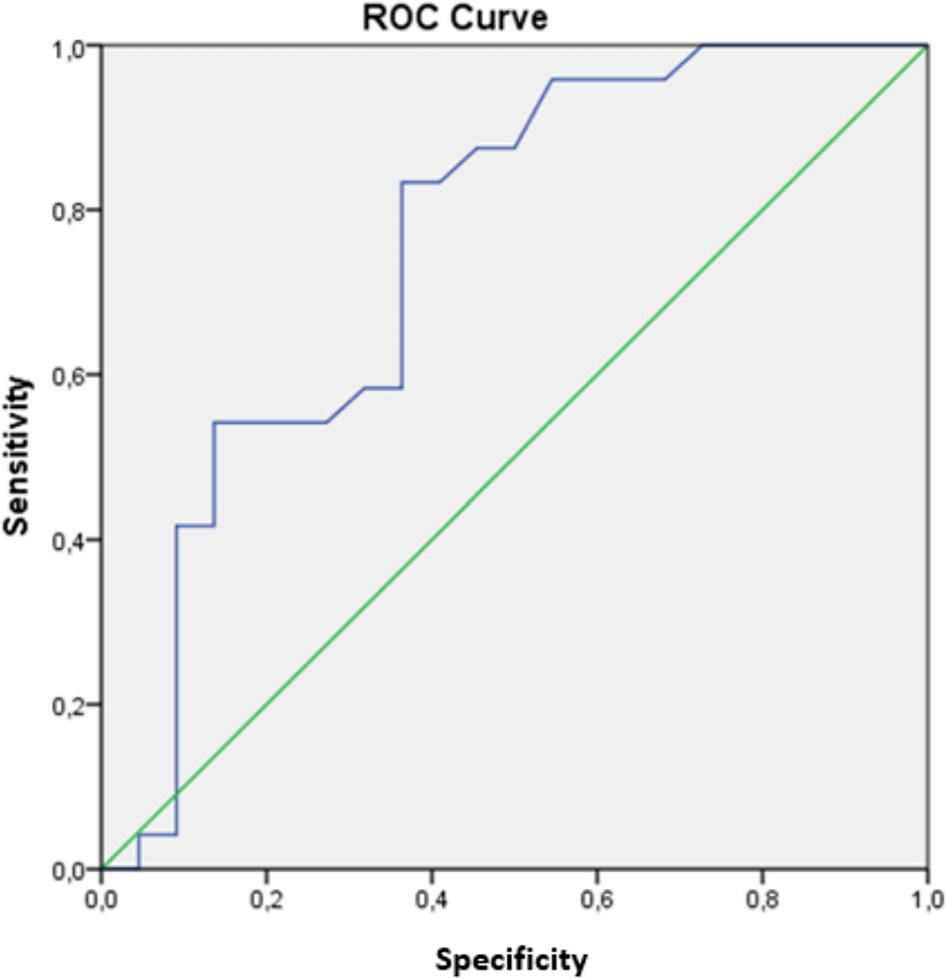
The graph for ROC analysis [*p* = 0.003 (ROC–AUC: 0.753; 95% CI: 0.606–0.899)]

The Receiver Operator Characteristics (ROC) was performed in the study for determining the differentiation strength of miR-218-5p expression level of sarcoma patients from the healthy patients. This analysis showed that the differentiation strength of the higher miR-218-5p expression level of sarcoma patients from healthy people was statistically significant (*p* = 0.003). (Fig. 3).

## STRING Analysis of miR-218-5p

The miR-218-5p and their target genes investigated in the present study were determined using the “mirTarBase” (https://bio.tools/mirtarbase) and “TargetScan” databases.. After obtaining a gene list from TargetScan, further refinement was carried out using the miRTarBase. These additional steps helped enhance the specificity of the gene targets associated with miR-218-5p. The STRING database facilitates the visualization of protein–protein interaction networks, enabling researchers to examine how the identified genes interact with each other at the protein level. This visualization aids in understanding the complex interplay between the gene targets associated with miR-218-5p, providing insights into their functional relationships and potential implications in biological processes.

**Fig. 4:**
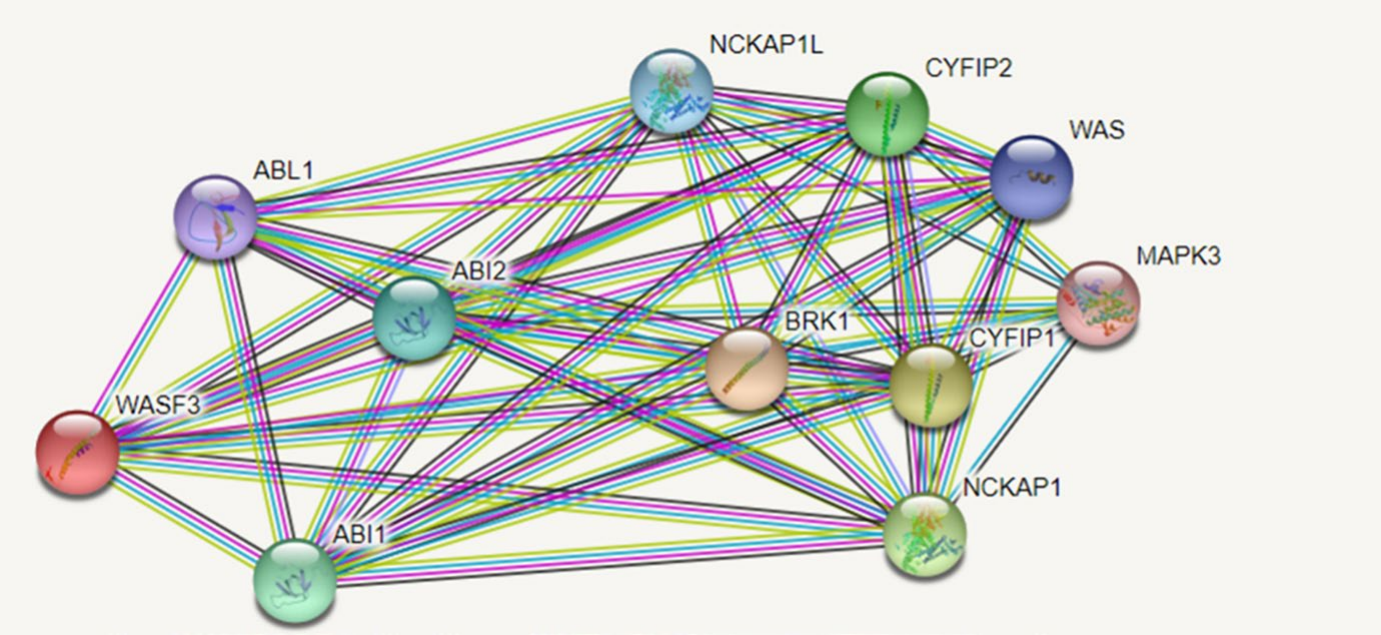
The STRING Analysis performed for the target genes of miR-218-5p (*p* value:1 × 10^–6^) (https://string-db.org/)

The “STRING Analysis (Protein–protein interaction analysis)” was performed for demonstrating the protein network of which the target genes of the miR-218-5p were mostly associated (Fig. 4). A total of 1084 genes including *WASF3, ABI1, ABI2, WIPF2, WASL,* and *ACTR3* were detected among the target genes. The protein level interaction results of the common gene targets detected for WASF3 using the STRING database were found significant (*p* = 1 × 10^–6^). The results of the STRING analysis are shown in Fig. 4. The target genes of the *WASF3* were found as the *ABL1, ABI1, ABI2, CYFIP1, CYFIP2,* *NCKAP1, NCKAP1L, WAS, MAPK3, BRK1* and *WASF3* genes. The protein interactions in the network obtained after the STRING analysis showed statistical significance (*p* < 0.05). The results of the STRING analysis showed that the genes targeted by miR-218-5p and their associated proteins the protein interaction was strong between *WASF3* and *ABI1*, and this association was found statistically significant (*p* = 0.0059).

## CONCLUSION

miRNAs have been identified as effective and strong regulatory molecules both in normal development and in cancer formation. Dysfunction of miRNAs in various cancers suggests that miRNA expression could be used as a new therapeutic approach for such diseases (Iorio and Croce 2012; Kumar et al. 2008). The miR-218-5p was reported to act as a tumor suppressor in many human cancers such as hepatocellular carcinoma, bladder, mouth, stomach, and retinoblastoma cancers (Tu et al. 2014; Li et al. 2021, 2020; Wang et al. 2020; Tang et al. 2016). In one of the studies, the expression level of miR-218-5p was shown to have significantly downregulated in breast cancer samples in breast cancer tissues compared to the expression levels in the normal breast tissues (Xia et al. 2019). Similar results have been reported in the studies conducted in cancer cell strains (Luu et al. 2017). In a different study, miR-218-5p was found to have supported the *ERBB2* and *EGFR* expression by inhibiting the *LRIG1* in breast cancer cells, which shows that miR-218-5p, and *LRIG1*can act as an oncogene in breast cancer and can be used as a therapeutic target for breast cancer treatments (Qian et al. 2021). In different cancers such as bladder, intraoral, and hepatocellular carcinoma, miR-218-5p expression level was found to be low and acts tumor suppressor, on the contrary, the expression level was found to be high in sarcoma cases in our study. This finding shows that the miR-218-5p acts oncogene in pediatric sarcomas.

The highest difficulty in studies conducted with sarcomas is that randomized trials cannot be conducted because these cancers have an extremely low incidence and are a heterogeneous group of diseases. The statistically significant expression level of miR-218-5p in patient groups compared to the levels in healthy controls (*p* = 0.006) suggests that miR-218-5p is a diagnostically and prognostically important molecule in sarcomas. The ROC analysis results which shows the differentiation strength of miR-218-5p molecule of sarcoma patients from health individuals supports this result with a statistical significance (*p* = 0.003). However, the analysis shows that the miR-218-5p is an effective molecular mechanism in the oncogenesis of sarcomas, and both miR-218-5p and the proteins with which this molecule interacts should be further investigated in future studies.

In our study, the expression level of miR-218-5p in the peripheral blood of a 16-year-old patient with osteosarcoma was investigated, starting from the diagnosis and during the different stages of treatment for the first time, and the expression level of miR-218-5p in peripheral blood was shown to have varied during treatment depending on the treatment. This change and the follow-up of miR-218-5p during treatment can be used to monitor the disease and determine the prognosis. In addition, obtaining of very close values with the miR-218-5p expressions in the peripheral blood of the patient while the resection of tumor tissue showed that the investigation of the molecule in the peripheral blood was as valuable as investigation in the tumor tissue, and peripheral blood samples may be used instead of the tumor tissue in this group of patients. However, the demonstration of these findings in large patient groups where a greater number of patients are followed-up will increase the value of this finding. All these results suggest that miR-218-5p may be both a diagnostic and prognostic non-invasive biomarker.

The STRING analysis revealed that there were much more significant interactions for miR-218-5p than the expected. Various genes such as *ABI1, ABI2, and WASF3* that have roles particularly in the regulation of the cell morphology and cell skeleton organization were shown among the target genes of miR-218-5p. The *ABI1* gene was shown to have played a role in tumor invasion and metastasis in breast cancer in a study conducted in 2021 (Regua et al., 2021). *WASF3* expression has been reported to have positive correlation with poor prognosis in stomach cancer patients (Nie et al. 2019). Such an interaction detected for miR-218-5p indicates that the proteins are at least partially biologically connected as one group and are associated with invasion.

The expression level of miR-218-5p, which was investigated in the blood and tumor tissue within the scope of the study showed that it has a high diagnostic value in tissue in Ewing’s sarcoma, osteosarcoma, and may be both a therapeutic and prognostic marker. Since they are rare tumors, the small number of patients constitutes the limitation of the study. Another difficulty in the study was that most patients lived out of Istanbul city, and pursued their treatment in the hospitals of their hometown after surgical procedure. Therefore, no peripheral blood samples during treatment and follow up could be obtained.

In conclusion, miRNAs have the potential to be promising therapeutic targets in sarcomas due to their critical role in various biological processes. Our study results suggest that the higher miR-218-5p expression in patients with sarcoma before treatment acts as an oncogene. In the study, the expression level of miR-218-5p changed depending on the treatment in the peripheral blood sample examined at the time of diagnosis, during the operation, and after the 1st and 4th cycles of chemotherapy in a patient with osteosarcoma, suggesting that this change can be used in the follow-up of a treatment and in determining the prognosis.

## Ethics Approval

The ethics committee approval of the study was obtained from the Clinical Research Ethics Committee in Istanbul University, Istanbul Faculty of Medicine dated 02.07.2020 with nu: 74742.
